# Deep carbon recycling viewed from global plate tectonics

**DOI:** 10.1093/nsr/nwae089

**Published:** 2024-04-12

**Authors:** Maoliang Zhang, Sheng Xu, Yuji Sano

**Affiliations:** School of Earth System Science, Tianjin University, Tianjin 300072, China; School of Earth System Science, Tianjin University, Tianjin 300072, China; Marine Core Research Institute, Kochi University, Kochi 783-8502, Japan; Atmosphere and Ocean Research Institute, The University of Tokyo, Chiba 277-8564, Japan

**Keywords:** deep carbon recycling, convergent plate margins, subduction, continental reworking, global carbon mass balance

## Abstract

Plate tectonics plays an essential role in the redistribution of life-essential volatile elements between Earth's interior and surface, whereby our planet has been well tuned to maintain enduring habitability over much of its history. Here we present an overview of deep carbon recycling in the regime of modern plate tectonics, with a special focus on convergent plate margins for assessing global carbon mass balance. The up-to-date flux compilation implies an approximate balance between deep carbon outflux and subduction carbon influx within uncertainty but remarkably limited return of carbon to convecting mantle. If correct, carbon would gradually accumulate in the lithosphere over time by (i) massive subsurface carbon storage occurring primarily in continental lithosphere from convergent margins to continental interior and (ii) persistent surface carbon sinks to seafloors sustained by high-flux deep CO_2_ emissions to the atmosphere. Further assessment of global carbon mass balance requires updates on fluxes of subduction-driven carbon recycling paths and reduction in uncertainty of deep carbon outflux. From a global plate tectonics point of view, we particularly emphasize that continental reworking is an important mechanism for remobilizing geologically sequestered carbon in continental crust and sub-continental lithospheric mantle. In light of recent advances, future research is suggested to focus on a better understanding of the reservoirs, fluxes, mechanisms, and climatic effects of deep carbon recycling following an integrated methodology of observation, experiment, and numerical modeling, with the aim of decoding the self-regulating Earth system and its habitability from the deep carbon recycling perspective.

## Teaser

This review examines how carbon is transferred between Earth’s deep and surface reservoirs based on an up-to-date carbon flux compilation for global plate tectonic settings and carbon mass balance assessment. It particularly recognizes the role of tectonic degassing driven by continental reworking in deep carbon recycling that is complementary to volcanic degassing from mid-ocean ridges, arcs, and plumes.

## INTRODUCTION

Volatiles (hereafter referring to noble gases and life-essential volatile elements or compounds of H, C, N, O, S, and halogens) and their recycling between Earth's deep and surface reservoirs through geological time are crucial to the co-evolution of habitability and life on our planet [1]. Since the emergence of the earliest liquid water in the surface or near-surface environment ∼4.3 billion years ago [2], the Earth has maintained habitable conditions although at times extreme glaciation or greenhouse climates prevailed and exerted variable extents of impact on the evolution of life [3]. The secrets behind the habitable Earth lie in the well-tuned cycles of carbon and other life-essential volatile elements [1]. It has been widely accepted that volatile recycling between the deep and surface reservoirs is dominated by ancient and modern plate tectonics that initiated in the Archean and Neoproterozoic, respectively [4–6], with episodic perturbations by short-lived but catastrophic eruptions of large igneous provinces (LIPs) [7]. Earth's long-term [>1 million year (Myr)] climate depends on the stability of steady-state source-and-sink feedbacks of atmospheric CO_2_ [8], in which the volcanic and metamorphic CO_2_ inputs to the atmosphere are consumed in equivalent amount by surface sinks via carbonate precipitation and organic carbon burial [9]. This sets a basis for the habitable surface environment over much of the geological history, highlighting that the interaction between deep and surface carbon cycles acts as the Earth's long-term climate modulator [10].

**Figure 1.**
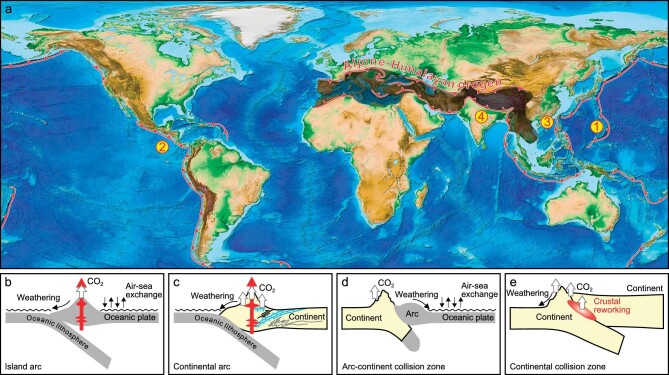
Modern-style global plate tectonics (a) and examples of convergent plate margins (b, island arc; c, continental arc; d, arc-continent collision zone; e, continental collision zone; modified from [14]). For the latter two types of convergent margins, the continental lithosphere, although relatively buoyant, could subduct to great depths and form a mantle wedge that resembles an oceanic subduction zone [15]. Global relief map in (a) is based on National Oceanic and Atmospheric Administration (NOAA) ETOPO1 1 Arc-Minute Global Relief Model [127].

In the regime of modern plate tectonics (Fig. 1a), convergent plate margins are the only sites that could transport surface volatiles back to the mantle [6], while the mid-ocean ridges (MORs), plumes, and intra-continental settings are considered as unidirectional pathways that allow volatile outgassing from the solid Earth to its fluid envelope (without direct replenishment to the mantle). Convergent plate margins, as classified based on the types of plates that converge together (Fig. 1b−e), are thus important for the mass balance assessment of volatile recycling between the mantle and surface [e.g. 5,6,11]. Looking back at the geological past, volatile element recycling in convergent plate margins has played a pivotal role in the deep-to-surface processes that shaped the Earth into its present-day state. One example is the volcanic CO_2_ released from continental arcs that may have driven climate swings between greenhouse and icehouse conditions since as early as ∼720 Myr ago [12]. The addition of aqueous fluids (mainly H_2_O and CO_2_) from the subducting slab promotes partial melting of the mantle wedge and formation of volatile-rich arc magmas [13], which could release large amounts of CO_2_ to the atmosphere especially when interacting with the crustal carbonate sequences in active continental margins [14]. Volatile recycling also contributes to mountain building in convergent plate margins. Accompanied with significant deformation and metamorphism during convergence (subduction or collision; [15]), the presence of volatiles in the melting zone (ranging vertically from sub-arc mantle wedge to arc crust) of convergent plate margins facilitates voluminous magma production [13] and thus the growth of continental crust [16] and mountain belts [17]. This is exemplified by the North American Cordillera (i.e. orogeny in oceanic subduction zone) and the Himalayas and Tibetan Plateau (i.e. orogeny in continental collision zone). In return, the mountain belts could modulate regional to global climate through a series of carbon cycle feedbacks (e.g. weathering of silicate, carbonate, and sulfide [18]; erosion and burial of rock + biospheric organic carbon [19]), changes in land-sea distribution [20], and reorganization of river drainage systems [21]. The integrated mountain building-related processes are fundamental to nutrient cycling from continents to oceans and thus the evolution of life [e.g. 22,23]. Overall, convergent plate margins link up the deep and surface Earth systems, dominate the long-term recycling of volatile elements (especially carbon), and are central to the stabilization of the physico-chemical basis that supports Earth's habitability.

The topic of volatile recycling is extremely broad considering (i) the whole family of noble gases and life-essential volatile elements [5], (ii) spatial heterogeneity in volatile reservoirs and fluxes under the context of modern global plate tectonics [6], and (iii) temporal evolution of the volatile recycling over time [24]. Several excellent reviews have been devoted to Earth's volatile origin, accretion, and degassing history [e.g. 25], noble gas systematics [e.g. 26], global mass balance in volatile recycling [e.g. 5], and the recycling of some specific volatiles (e.g. H_2_O, C, and N; [e.g. 6,11,27]). These reviews, as well as those omitted unintendedly due to the limit of our knowledge, provide important insights into the reservoirs, fluxes, and mechanisms of volatile recycling in the regime of global plate tectonics. For simplicity and clearance, our focus here is carbon, one of the most important life-essential elements with its compound CO_2_ essential for global climate changes.

In light of recent advances, we conceive this review with rethinking on reservoirs, fluxes, and mechanisms of deep carbon recycling from a viewpoint of present-day global plate tectonics, which particularly highlights the role of continental reworking as a globally significant mechanism of deep CO_2_ emissions. Our motivation is quite definite because most previous studies center on the MORs, arcs (including island arcs and continental arcs), and plumes that exhibit close affinity with oceanic plates (see detailed review by Bekaert *et al*. [5]). Besides that, tectonic CO_2_ emissions in continental regions, where active volcanoes could also exist (although much sparser than MORs, arcs, and plumes), have received increasing attention, as shown by observations in continental rifts [e.g. 28,29] and collisional orogens [e.g. 30,31]. Owing to the growing database of carbon fluxes in both oceanic and continental settings, a further assessment of global carbon mass balance is thus feasible for the convergent plate margins. In this review, we first briefly summarize the deep and surface reservoirs that participate in deep carbon recycling, in which noble gases and other volatile elements are also discussed in order to understand the origin of Earth's carbon. Then, a following section is devoted to the pathways and mechanisms of deep carbon recycling following the theory of global plate tectonics. A flux-based assessment of global carbon mass balance is further presented. At the end, we put forward the grand challenges and some potential research opportunities in deep carbon recycling. Taken together, we aim to achieve a balance between the completeness of coverage and new insights for future research.

## EARTH’S VOLATILE RESERVOIRS FROM DEEP TO SURFACE

### Defining the deep and surface reservoirs

**Figure 2.**
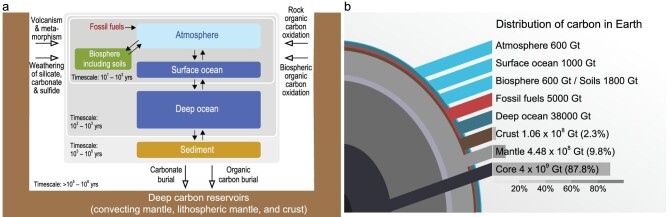
Deep and surface reservoirs and carbon recycling processes (a; modified from [125]) and carbon budgets of each reservoir (b; modified from a cartoon by Josh Wood). Data of carbon budgets are from refs [27, 33, 48]. Numbers in brackets represent mass fraction of carbon in the core, mantle, and crust relative to Earth's total carbon budget.

Earth's volatile reservoirs refer to the multiple spheres (mainly including the core, mantle, crust, hydrosphere, atmosphere, and biosphere) ranging from its interior to the surface. The present-day volatile budgets of the multiple spheres result from accretion, loss, recycling, and redistribution of volatile elements over time [5]. Thus, defining the interface between the deep and surface reservoirs is needed to clarify the interaction between them and to elucidate how deeply-sourced volatiles (e.g. CO_2_) could impact Earth's surface environment. A prevailing classification for understanding volatile recycling paths focuses on Earth's interior (i.e. mantle) and exosphere (i.e. atmosphere, ocean, and crust) [e.g. 27,32] and the latter is interpreted as a reservoir to host the total amount of volatiles degassed throughout Earth's history [32]. However, considering that the continental and oceanic crust is a long-term reservoir and source for volatile elements such as carbon, we adopt a classification scheme that consists of endogenic and exogenic systems in this review (Fig. 2a). Specifically, the endogenic system refers to the deep reservoirs (defined here as the combination of convecting mantle, lithospheric mantle, and crust), while the exogenic system represents the surface reservoirs composed of atmosphere, oceans, reactive marine sediments, and terrestrial biosphere including soils [8]. Note that the core is an important reservoir, and for example, the mass of carbon in the core is ∼4 × 10^9^ Gt [33], which constitutes about 88% of the Earth's carbon (and ∼8 times more than is in the mantle; Fig. 2b). Although some recent studies suggest a core contribution to solid Earth degassing [e.g. 34], the core will not be focused on here due to its less quantitatively constrained exchange with the mantle [5].

### Convecting mantle

**Figure 3.**
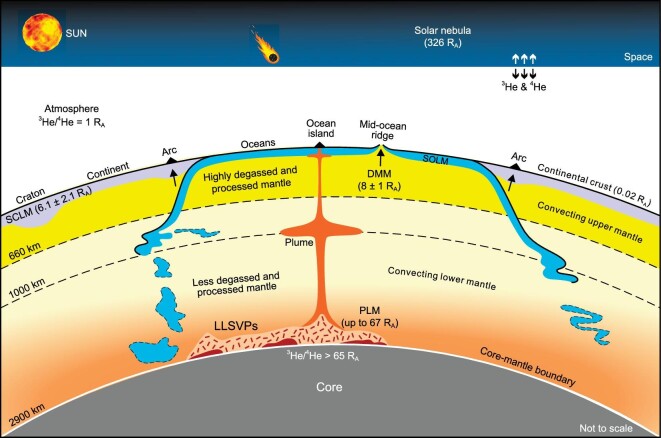
Earth's deep and surface volatile reservoirs with ^3^He/^4^He ratios shown for information (modified from [26]). Abbreviations: PLM, primitive lower mantle; DMM, depleted mid-ocean ridge basalt (MORB)-source mantle; SCLM, sub-continental lithospheric mantle; SOLM, sub-oceanic lithospheric mantle; LLSVPs, large low-shear-wave-velocity provinces. Data sources of ^3^He/^4^He ratios are as follows: solar nebular, atmosphere, and continental crust (Ozima and Podosek [46]); SCLM (Day *et al*. [47]); DMM (Graham [36]); PLM and the core (Horton *et al*. [34]).

The convecting mantle includes primitive lower mantle (PLM) and depleted mid-ocean ridge basalt (MORB)-source mantle (DMM) (Fig. 3), which together with the rigid lithospheric mantle accounts for ∼99.4% of bulk silicate Earth by mass [35]. As Earth's largest layer, the convecting mantle is an important reservoir for primordial volatiles, i.e. those incorporated primarily during Earth's main accretion phase from three potential sources, including solar nebula gas, solar-wind-irradiated meteoritic materials, and chondritic meteorites [25,26]. Mantle-derived basalts [e.g. MORBs and ocean island basalts (OIBs)] and free gases from active (or quiescent) volcanoes and continental drilled wells preserve key information on the origin of Earth's volatiles [e.g. 36–39]. For example, the capture of solar nebular gases in the early Earth is recorded by ^20^Ne/^22^Ne ratios of some plume-influenced basalts (see [26] and references therein) and D/H ratios of the Baffin Island and Icelandic lavas [40]. Notably, the Ne isotopes also suggest the presence of solar-wind-irradiated meteoritic materials [37] in the MORB source, or alternatively, mixing of solar nebular gases with atmospheric Ne or CI chondritic materials [26]. It is believed that most of the nebular volatiles acquired by the proto-Earth embryo (1–3 times the size of Mars) were lost and replaced with later-accreted volatiles [25], as evidenced by (i) the chondrite-like Ar, Kr, and Xe isotopic signatures preserved in MORBs and/or OIBs [26] and (ii) major volatiles (e.g. H, C, and N) that exhibit chondritic signatures [41].

Throughout Earth's history, the volatile inventory of convecting mantle has been processed by multi-stage partial melting and degassing (i.e. volatile transfer from convecting mantle to the surface), time-integrated radiogenic ingrowth of noble gas isotopes [26], and subduction-driven ingassing (i.e. the transfer of surface volatiles back to the mantle) [1]. Particularly, carbon in the present-day convecting mantle is low in concentration (e.g. 110 ± 40 ppm; [27]) but constitutes a large fraction (∼81%) in total carbon budget of the bulk silicate Earth (Fig. 2b). As shown in Fig. 3, the spatially heterogeneous mantle degassing is well documented by noble gas systematics of MORBs and OIBs. This provides the cornerstone for the ‘layered mantle’ model [42], in which the PLM represents a less degassed and processed portion of convecting mantle (e.g. the highest terrestrial ^3^He/^4^He ratio up to ∼67 R_A_ in the Baffin Island basalts [34], where R_A_ = air ^3^He/^4^He = 1.39 × 10^−6^) relative to the DMM (^3^He/^4^He = 8 ± 1 R_A_; [36]). Several models have been put forward to account for the contrasting ^3^He/^4^He ratios between the PLM and DMM, such as (i) convective isolation of mantle plume source and (ii) crystallized residue of dense melts in the lower mantle generated by deep and hot melting during the Earth's first billion years (see [26] and references therein). Additionally, the core has been suggested to be a potential source for ^3^He (Fig. 3; [34]) and large low-shear-wave-velocity provinces (LLSVPs) in the lowermost mantle are also invoked as candidates for the preservation of ^3^He and sources of mantle plumes [e.g. 43].

### Lithospheric mantle

The lithospheric mantle represents a significant portion of the upper mantle that is stabilized against convection beneath the continents and oceans (Fig. 3). It receives the uprising volatile-rich melts primarily in two ways: (i) gradual melt influx from the pervasive convecting upper mantle, and (ii) episodic melt influx from mantle plumes [44]. These melts could migrate through the lithosphere and release large amounts of magmatic volatiles (e.g. H_2_O and CO_2_) to the surface reservoirs especially during LIP events [7]. However, much of the melts are expected to react with peridotite or to be simply solidified as metasomatic veins in the lithospheric mantle [45]. Due to the complex metasomatism during its prolonged evolutionary history, ancient lithospheric mantle is temporally, spatially, and chemically heterogeneous at regional to global scales. Mantle-derived xenoliths and diamonds provide rare information on elemental and isotopic signatures of volatiles trapped in the lithospheric mantle [46]. For example, the sub-continental lithospheric mantle (SCLM) is commonly suggested to have average ^3^He/^4^He of 6.1 ± 2.1 R_A_ [47], but globally the possibility of helium isotope heterogeneity still exists. From the convergent plate margins [11] to continental interior [44], the SCLM could sequester considerable carbon in different forms (e.g. diamonds in cratonic keels; [44]) and act as a globally significant carbon source over geological timescales [48]. In the case of sub-oceanic lithospheric mantle (SOLM), the serpentinization of mantle peridotite could sequester considerable amounts of carbon in MORs, transform faults, fracture zones, and outer rise regions where the slab bends [11]. Recent work by Gibson and McKenzie [48] quantified major volatile budgets of the lithospheric mantle end-members and showed that bulk carbon concentration of the SCLM is higher than that of the SOLM by a factor of ∼3, consistent with the expected essential role of the SCLM as a deep carbon source [44].

### Crust

Continental crust and oceanic crust make up ∼0.6% of the bulk silicate Earth by mass [35]. They are the outermost layers of solid Earth, averaging in thickness of ∼40 km beneath continents and of ∼7 km beneath oceans [48,49], and contain about (1.06 × 10^8^ Gt C in total (Fig. 2b; [27,33]). The crust represents a transitional interface between the deep and surface reservoirs [8] and is the place for erosion, weathering, sediment deposition, and biological activity, by which large amounts of volatile elements are sequestered in rocks. Volatile elements in the crust are mostly bounded to minerals either as major components or as substitutes; and they are also preserved in organic matter [33]. A trivial yet important type of volatile-bearing phase in the crust is the H_2_O- and CO_2_-bearing fluids, including magmatic, metamorphic, and hydrothermal fluids occurring from deep crustal to shallow levels [50,51]. The most common crustal fluids are those circulating in groundwater systems that are exclusively hosted by the upper crust. Evidence for the presence of lower crustal fluids comes from deep-seated rocks and geophysical observations (see [50] and references therein). In the convergent plate margins, both continental crust and oceanic crust play major roles in transferring volatiles to deep crustal and mantle depths [13,52], thus facilitating slab-mantle interaction, return of the recycled volatiles to the surface, and storage of magma-derived volatiles in the overriding lithosphere (see details in Section 3.1).

### Surface reservoirs

Earth's transformation from a magma ocean planet in its very beginning to a habitable life-fostering world is determined by volatile accretion, loss, and redistribution over time between its solid layers, fluid envelope (i.e. atmosphere and oceans), and biosphere [1]. Solid Earth degassing, air-sea exchange, and escape into outer space (especially for He and H_2_) collectively modulate the volatile budgets of the atmosphere (Fig. 3). Noble gases could be stabilized and well mixed in the atmosphere under present-day surface conditions, with their exchange with the oceans governed by element-dependent solubility [26]. Life-essential elements (e.g. H, C, N, O, and S) in the surface reservoirs are cycling among its sub-systems (i.e. atmosphere, oceans, pedosphere, as well as the terrestrial and marine biosphere) in various ways and forms, i.e. the biogeochemical cycles that are beyond the scope of this review. Since the surface carbon budgets (∼4.2 × 10^4^ Gt C in total; Fig. 2b) are quite trivial compared to the deep carbon reservoirs, the episodic changes in endogenic CO_2_ fluxes due to intensive volcanism and metamorphism would lead to long-term feedbacks in surface carbon cycles and re-organization of Earth's self-regulation system [9,10]. Therefore, when discussing deep carbon recycling in the following sections, we will take the surface reservoirs and the rapidly operating biogeochemical cycles as a whole end-member.

## PATHWAYS AND MECHANISMS OF GLOBAL DEEP CARBON RECYCLING

The preceding section summarizes the Earth's deep and surface volatile reservoirs, and here we focus on how carbon is transferred between them. Considering their contrasting carbon budgets (Fig. 2b), we particularly emphasize the role of deep carbon emissions (volcanic and metamorphic CO_2_ outgassing) in disturbing the near steady-state carbon budget of the exogenic system [8], in which carbon is rapidly cycling over short-term timescales (e.g. <0.5 Myr; [14]). Following Plank and Manning [6], we refer to the return of endogenic carbon to the exogenic system as ‘recycling’, which is operating via two first-order mechanisms: subduction and continental reworking.

### Subduction-driven carbon recycling

**Figure 4.**
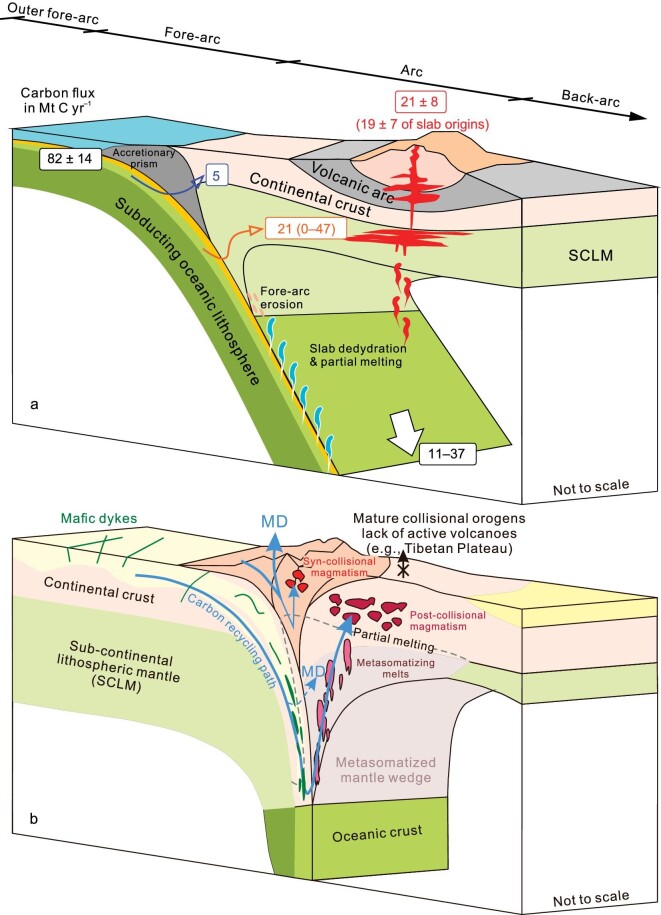
Cartoon models showing pathways and mechanisms of deep carbon recycling in the oceanic subduction zone (a) and the continental subduction zone (b; modified from [52]). Numbers in rectangular boxes of panel (a) represent carbon fluxes discussed in the main text. Deep and shallow levels of metamorphic decarbonation (MD) are shown in (b) for deep carbon (C) recycling in a continental subduction zone.

The most important mechanism of deep carbon recycling is subduction [6]. Fig. 4 shows the primary pathways and mechanisms of deep carbon recycling at oceanic subduction zone (e.g. continental arc) and continental subduction zone (e.g. collisional orogen). The pathway through which subducting materials enter the mantle wedge is termed as ‘subduction channel’, which was originally developed for oceanic subduction zones and has now been extended to continental subduction zones [53]. Different convergent margins generally share similar mechanisms of carbon recycling (e.g. slab devolatilization and partial melting, mélange diapirism, and mantle metasomatism; e.g. [54]) in the subduction channel, but could vary significantly in carbon recycling efficiency due to differences in (i) the assemblage of slab lithologies and sediments (i.e. bulk compositions), (ii) pH and redox potentials, and (iii) subduction-zone thermal regimes [5,6]. Carbon behavior along the subduction geotherms has been hotly studied by observational, experimental, and numerical work [e.g. 55–60]. Spatially, the carbon recycling paths at oceanic subduction zones could be interpreted from a cross-section that covers the outer fore-arc, fore-arc, arc, and back-arc regions (Fig. 4a), which is best exemplified by the Costa Rican convergent margin detailed in Barry *et al*. [55].

Outer fore-arc (defined here as the slab outer rise region prior to its arrival at the trench) has the capacity to sequester carbon in serpenitized peridotite (e.g. 0.6–2 Mt C yr^–1^ [61] or 4–12 Mt C yr^–1^ [11]) due to pervasive slab bending-induced hydration [e.g. 62]. Prior to arriving at sub-arc depths, carbon could be subtracted from subducting slab mainly through mechanical removal, metamorphic decarbonation, and partial melting [6]. For example, solid storage of carbon-bearing slab materials via mélange diapirs in the sub-arc lithosphere is an important mechanism for carbon removal from the subducting slab [11,57]. Previous studies show that carbonate dissolution and hydrous melting of alternated oceanic crust and sediments dominate the mobilization of subducting carbon at sub-arc depths [e.g. 60], providing CO_2_-rich melts for the arc volcanoes. As the major carbon-bearing phase in subducting slabs [6], carbonate could survive the sub-arc melting and reach the deeper mantle [56–58], such as the mantle transition zone where low-degree partial melts of the carbonated oceanic crust could react with the ambient mantle to produce diamonds [56]. Toward the back-arcs and continental interior, carbon recycling and outgassing remain less quantitatively constrained compared to the volcanic arc front [55]. Nevertheless, the deeply subducted carbon and its impact on mantle metasomatism and related partial melting, which commonly occur in big mantle wedges such as that observed beneath East Asia due to subduction of the western Pacific plate [63], are emerging as a globally significant research topic in deep carbon science [64–66].

Deep carbon recycling in the continental subduction zone (Fig. 4b) has been discussed in many studies [e.g. 52,67,68] but remains less quantitatively constrained than that driven by oceanic subduction. Unlike the island arcs and continental arcs, a primary feature of collisional settings is the transition from oceanic subduction to continental subduction [69], during which the closure of paleo-oceans (e.g. the Neo-Tethys Ocean) is followed by successive collision and subduction of incoming continents. Multi-stages of magmatism and metamorphism would take place in response to such plate convergence transition [e.g. 68,70]. The huge Alpine-Himalayan orogen (Fig. 1a) is a natural laboratory for studying deep carbon recycling related to the transition from oceanic to continental subduction [71]. For example, the Mediterranean regions represent remnants of the Tethys Ocean and the complex plate tectonics over time that involved subduction-related processes is recognized in magma and associated volatile outgassing from many active volcanoes, i.e. CO_2_ emitters such as Mount Etna and Vulture [72,73], in Italy and adjacent regions [74]. In contrast, active volcanoes are generally rare in continental collision zones from the Iranian Plateau to the Tibetan Plateau; but it has been noted that voluminous magma eruptions in the geological past (e.g. the Linzizong volcanism; [75]) may have caused global climatic impact. In particular, Guo *et al*. [68] calculated the fluxes of CO_2_ released by continental collision-related magmatism in the Tibetan Plateau and proposed that India-Asia collision could be a primary driver for the changes in atmospheric CO_2_ levels over the Cenozoic. As a characteristic type of magmatism in collisional orogens, the post-collisional potassium-rich magmas are suggested to have close affinity with the enriched mantle sources that were previously metasomatized by recycled carbon-bearing phases [e.g. 52,68]. Such a carbonated mantle source beneath the Tibetan Plateau has been proposed to account for present-day deep CO_2_ emissions where mantle fluid inputs are evident [76], suggesting carbon recycling paths that resemble those of the oceanic subduction zones.

### Carbon remobilization induced by continental reworking

**Figure 5.**
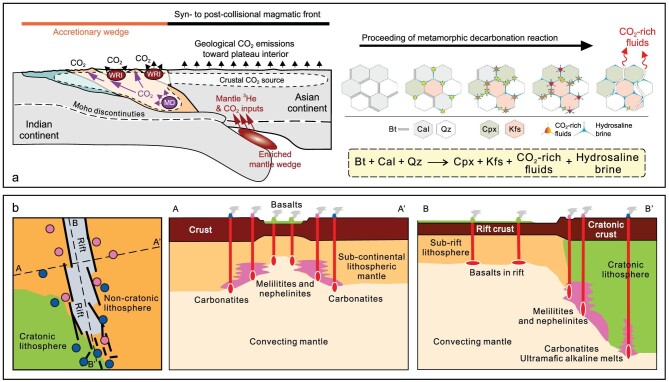
Cartoon models showing the reworking of continental crust (a; modified from [84,95]) and sub-continental lithospheric mantle (b; modified from [44]). Abbreviations: WRI, water-rock interaction; MD, metamorphic decarbonation. For collisional orogens represented by the India-Asia collision zone, the across-orogen profile defines a transition of geological CO_2_ emissions from accretionary wedge to (syn- and post-collisional) magmatic front [95]. Metamorphic decarbonation at different crustal depths could account for most of the CO_2_ origins, although a minor fraction of mantle CO_2_ inputs has been identified in hydrothermal systems of the magmatic front. In stark contrast, CO_2_ emissions from continental rifts are predominated by mantle carbon inputs due to partial melting of carbon-rich mantle sources, especially for the metasomatized SCLM in the margins of ancient cratons [29,44].

The reworking of continent is defined as the superposition of younger geological events onto the older geological systems (see comprehensive review by Zhu *et al*. [17]). Continental reworking commonly results in pervasive magmatism and metamorphism [17], which have the capacity to mobilize a substantial amount of mineral-bound volatiles (e.g. H_2_O and CO_2_) into melts and/or fluids [44,77] and to facilitate their upward transfer and outgassing through permeable conduits such as active faults [78,79]. Note that it is referred to as ‘reworking’ or ‘remobilization’ in this review, because prior to being remobilized, carbon has been sequestered in continental crust and the SCLM for durations far exceeding the turnover time of biogeochemical cycles [e.g. 14,52,72]. Moreover, it differs from the subduction-driven carbon recycling because subduction is not a prerequisite for the reworking of carbon stored in continental lithosphere, unlike the direct carbon recycling and transfer to the surface through arc volcanoes. Globally, continental reworking could occur in many tectonic settings [17], especially for (i) collisional orogens where crustal reworking is significant [80] (Fig. 5a) and (ii) continental rifts characterized by the reactivation of the SCLM [29,44] (Fig. 5b). In addition, the remobilization of crustal carbon caused by magma intrusion also occurs at continental arcs [e.g. 14,81]. Taken together, the reworking of continent-hosted carbon is a globally significant carbon recycling mechanism, which has been highlighted in recent studies on carbon remobilization and outgassing, as well as related climatic impacts [e.g. 44,82,83].

Continental collision-related magmatism and metamorphism are the first-order mechanisms for carbon remobilization in collisional orogens such as the Himalayas and Tibetan Plateau [e.g. 68,70]. Considering that mantle-derived volcanism is weak in modern collisional orogens (e.g. the cease of post-collisional volcanism in southern Tibet since ∼8 Myr ago; [69]), the subduction-driven carbon recycling processes are less significant for active orogenic CO_2_ emissions at present. Instead, metamorphic decarbonation [e.g. 70,82,84] and crustal anatexis [e.g. 52,77] have been suggested to account for most of the present-day CO_2_ outfluxes in collisional orogens (Fig. 5a). In cases of both crustal anatexis (e.g. >700°C) and metamorphic decarbonation reactions (e.g. 300 to < 650 − 700°C), the presence of crustal fluids in the source is critical for carbon remobilization [77,82,84]. Metamorphic rocks exhumed from depths greater than ∼15 km provide the robust evidence (e.g. melt/fluid inclusions that are in support of volatile storage in lower crust [77]) for the contributions of fluid phases to metamorphism and magmatism [60]. Crustal fluids are highly mobile and reactive, vary in composition under different P-T conditions, and play major roles in mobilizing soluble components from the country rocks (e.g. carbonate dissolution; [85]). However, fluid-rock reactions following prograde metamorphic devolatilization could also lead to carbon re-sequestration from the CO_2_-bearing fluids as carbonate [86] and/or graphite [84], which could thus hamper the transfer of remobilized carbon to the atmosphere. Moreover, the efficiency of carbon remobilization and CO_2_ outgassing would greatly increase when (i) anatexis of carbonate-bearing sediments occurs and generates CO_2_-rich melts [77], and/or (ii) anatectic melts intrude and interact with the crustal carbonate rocks. Overall, young and hot collisional orogens (e.g. the Himalayas; [87]) are important sites for crustal reworking and carbon remobilization.

We note that reworking of the SCLM in continental collision zones are still less understood with respect to its role in the deep carbon cycle. In contrast, continental rifting or breakup has been highlighted for its essential role as an important mechanism of deep carbon emissions [44], which probably dominated the Mesozoic and early Cenozoic greenhouse climate [83]. Particularly, the interaction zone between ancient cratons and orogens (Fig. 5b), such as the Tanzanian craton and the East African rift that are influenced by extensional tectonics ([29, 44]), could be viewed as the targeted study areas for understanding the SCLM reworking and related deep carbon emissions. This may be informative for exploring the mechanism of deep carbon recycling through the genesis of mantle-derived, volatile-rich magmas (e.g. carbonatites and kimberlites) in collisional orogens [e.g. 67,74] and the margins of ancient cratons [e.g. 88,89].

## MASS BALANCE ASSESSMENT FOR GLOBAL DEEP CARBON RECYCLING

After introducing the pathways and mechanisms of deep carbon recycling, we now focus on the assessment of global carbon mass balance. We note that comprehensive quantification of deep carbon recycling has recently been conducted in excellent reviews by, for example, Kelemen and Manning [11], Plank and Manning [6], and Bekaert *et al*. [5]. As such, we have simply compiled deep carbon outflux from global plate tectonic settings and subduction carbon influx (see details in Supplementary Data), which allow us to present a flux-based assessment of global carbon mass balance. The uncertainty of such assessment is illustrated in the following sections.

### Subducting carbon into and out of the mantle

Previous estimates of total subduction carbon influx vary significantly from ∼41 to 61–279 Mt C yr^–1^ (Supplementary Data). Here we adopt a recent estimate 82 ± 14 Mt C yr^–1^ by Plank and Manning [6] as the subduction carbon influx for carbon mass balance assessment at arcs (Fig. 4a). A small fraction of carbon in the subducting slab could be released by submarine vents in the fore-arc region [90], resulting in <1% loss of the subducting carbon via outgassing (0.66 + 0.58/–0.65 Mt C yr^–1^) if simply extrapolating the Costa Rican fore-arc carbon outgassing fluxes [55,90] to global active arcs (total length = 41,048 km; [5]). Barry *et al*. [55] found that the most important mechanisms responsible for fore-arc carbon removal from the subducting slab are carbonate precipitation and microbial chemolithoautotrophy. Their results show that most of the carbon released in the fore-arc region could be sequestered as calcite (∼91%) and biomass (∼3%), which corresponds to a simply extrapolated carbon storage flux of 9.8 ± 9.6 Mt C yr^–1^ at global fore-arcs (Supplementary Data). Note that the uncertainty of such scaling up is large due to the heterogeneity in carbon recycling efficiency among global arcs [6,71]. The results of Barry *et al*. [55] further suggest a reduction of subduction carbon influx (up to 19%) to the mantle beneath the Costa Rican convergent margin. This is clearly a pervasive signature for global subduction zones. For example, recent thermodynamic modeling indicates that ∼40% to 65% of the carbon in typical subducting crust could be mobilized through metamorphic decarbonation reactions at fore-arc depths [59], resulting in limited carbon transfer to the deep mantle. An earlier yet lower estimate of the fore-arc carbon storage (0.2–1.3 Mt C yr^–1^) is available in Kelemen and Manning [11]. We speculate that a range of 1–10 Mt C yr^–1^ or an average of ∼5 Mt C yr^–1^ is likely for carbon outgassing and storage at the fore-arcs (Fig. 4a), but further tests are required.

A recent study by Chen *et al*. [57] modeled the massive solid storage of subducting carbon into the sub-arc lithosphere, with the estimated flux ∼21.4 Mt C yr^–1^ agreeing well with average of a previous estimate ranging from 0–47 Mt C yr^–1^ [11]. Average carbon outflux from arc volcanoes is about 21 ± 8 Mt C yr^–1^ (1σ, *n* = 19), which is comparable with the MOR carbon outflux (23 ± 13 Mt C yr^–1^ (1σ, *n* = 37) (Table 1). In contrast, the back-arc carbon outflux remains poorly known. Also unclear is how much subducting carbon could be transported to sources of back-arc magmas, which could be a mixture between slab-derived materials and the ambient mantle [91]. Due to the paucity of carbon fluxes, we simply assume null (or uncertain) subducting carbon transport to the back-arc regions, and therefore, will not consider it in the assessment of subduction-related carbon mass balance (Fig. 4a). Clearly, future quantification of the subducting carbon into and out of the back-arcs would reduce the uncertainty in this mass balance assessment.

**Table 1.** Major plate tectonic settings with a summary of their present-day deep carbon outfluxes, pathways of carbon outgassing, and carbon sources.

Tectonic settings	Carbon outflux (Mt C yr^–1^)^a^	Pathways of carbon outgassing	Carbon sources
Mid-ocean ridges	23 ± 13 (1σ, *n* = 37)^b^	Active mid-ocean ridge volcanoes, including submarine hydrothermal systems	Convecting mantle, DMM, net mantle carbon sources
Plumes	9 ± 6 (1σ, *n* = 9)^c^	Active plume or hotspot volcanoes (i.e. ocean islands and seamounts), including hydrothermal systems	Convecting mantle, PLM, net mantle carbon sources
Arcs	21 ± 8 (1σ, *n* = 19)	Active arc volcanoes (island arcs and continental arcs), including hydrothermal systems	Subducting slab, mantle wedge, and overlying lithospheric mantle and crust of the main arc
Collisional orogens	26 (17–36)	Active tectonic CO_2_ degassing in collisional orogens	Mainly of crustal origins, locally showing low fractions of mantle carbon
Continental rifts	17 (8–25)	Active tectonic CO_2_ degassing in continental rifts	High fractions of mantle carbon, and crustal carbon could also exist
Total	96 ± 45	–	–

a Flux values (reported in Mt C yr^–1^) with 1SD (σ) are statistical results based on the compiled dataset of global deep carbon outflux (see full list of carbon outfluxes in the Supplementary Data). ^b^Flux estimates >100 Mt C yr^–1^ were excluded from calculation of average carbon outflux from mid-ocean ridges due to their large deviation from the majority of mid-ocean ridge data. ^c^A baseline flux is assumed for the modern plume volcanoes considering that (i) they are expected to have lower carbon outflux by several orders of magnitude at present than in active periods, and (ii) several studies suggest a much smaller carbon outflux for modern plume volcanoes than mid-ocean ridge fluxes (e.g. [105]).

The compiled data show that arc volcanoes account for ∼26% ± 10% of total subduction carbon influx, in good agreement with the result of Plank and Manning (27% + 23%/–16%; [6]). Marty and Tolstikhin [92] suggested that ∼80% of carbon released by arc volcanoes is derived from the subducting slab, and Kagoshima *et al*. [93] recently revised this value to 80%–95% (average 89%). Following the latter value, the arc carbon outflux of subduction origins would be ∼18.7 ± 7.1 Mt C yr^–1^, with the rest accounted for by mantle carbon (∼2.3 ± 0.9 Mt C yr^–1^). The carbon recycling efficiency at arcs, defined here as the fraction of subducting carbon to the surface reservoirs, may thus have a range of 23% ± 9%. Note that this value is obtained from simple calculation and there is no single carbon recycling efficiency for global arcs as stated in Plank and Manning [6]. For the continental subduction zones (Fig. 4b), the deep carbon recycling paths are far less quantitatively constrained than those done for arcs, although estimates of subducting carbon inventory have been thoroughly reviewed for global collisional settings such as the Alpine-Himalayan orogen [71].

### Mass balance assessment for the convecting mantle

**Figure 6.**
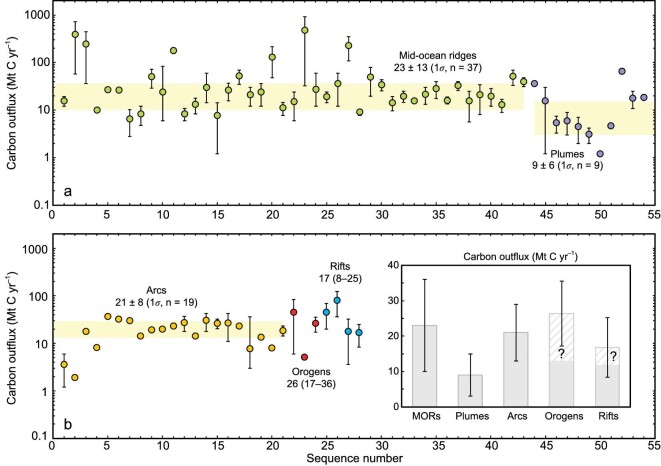
Diagram showing carbon outfluxes (Mt C yr^–1^) for mid-ocean ridges (MORs) + plumes (a) and arcs + orogens + rifts (b). Full list of the compiled data and references are available in the online Supplementary Data. An inset in (b) is shown for the best estimates (and upper/lower limits) of carbon outfluxes from global plate tectonic settings. Diagonal ruling and question mark denote the uncertain fractions of shallow carbon contributions to the total carbon outflux.

Unlike arcs, MORs and plumes represent net sources of mantle volatiles. They are the locus of most of the present-day mantle degassing [41], which are followed second by arcs as shown by ^3^He flux estimates [e.g. 5]. Therefore, comparing the sum of mantle carbon outfluxes from MORs, plumes, and arcs with the subducting carbon return flux to the convecting mantle would give an assessment for mass balance of the convecting mantle. The average carbon outfluxes from MORs and plumes are ∼23 ± 13 and 9 ± 6 Mt C yr^–1^ (Fig. 6a and Table 1), respectively. Together with mantle carbon outflux from arc volcanoes, mantle carbon (or primordial carbon) may be outgassing from the convecting mantle at a rate of ∼34 ± 20 Mt C yr^–1^. Previous studies suggested variable amounts of subducting carbon into the convecting mantle, ranging from 0 to 52 Mt C yr^–1^ (Supplementary Data; [11,57]). Several lines of evidence indicate that there might be limited replenishment of the convecting mantle by subducted carbon (e.g. <10 Mt C yr^–1^; [11,57]). In this review, the compiled data show that carbon return flux to the convecting mantle is about 11–37 Mt C yr^–1^ (Supplementary Data), with the lower and upper limits determined by the selection of sub-arc carbon storage (i.e. 21 vs. 47 Mt C yr^–1^). Taking all of the carbon recycling paths together, it is possible that the mantle carbon outflux remarkably exceeds the surface carbon influx to the convecting mantle, especially if considering a limited return flux of subducting carbon <10 Mt C yr^–1^ [57]. Moreover, mantle fraction of the carbon outflux in continental settings (mainly referring to continental rifts; [28]) would turn this imbalance to an even higher extent. And if such an imbalance stands, the convecting mantle would decrease in carbon budget over geological timescales [11,59]. However, it is also noted that recent quantification of the global carbon recycling points to net ingassing of carbon to the convecting mantle [5]. Therefore, we suggest that uncertainties in modern carbon inputs to and outputs from the convecting mantle are still (and inevitably) large at present. Further refinement of calculations is needed to resolve the ongoing debate on carbon mass balance or imbalance of the convecting mantle [e.g. 5,11].

### Role of continental reworking in deep carbon recycling

It is widely accepted that continental lithosphere, including continental crust and the SCLM, is an important carbon reservoir due to its capacity to sequester a massive amount of carbon in convergent plate margins (i.e. subducting carbon storage; [11,55,57,59]) and continental interior (i.e. influx of carbon-rich melts and mantle metasomatism; [44,48]). Also, the continental crust hosts ∼7.6 × 10^7^ Gt C in sedimentary rocks and shelf sediments [27]. This sedimentary carbon is mainly stored in the upper crust and could also be introduced to lower crustal depths during continental underthrusting (e.g. accretion of limestone platforms; [14]). Moreover, subsurface calcite precipitation is a globally pervasive hydrothermal process during the uprising of CO_2_-rich fluids [51,55,86], which could sequester considerable deep carbon at shallow levels. The huge amounts of continent-hosted carbon and its high potential to be remobilized collectively enhance the significance of continental lithosphere as a source of deep carbon, as highlighted in related studies on continental rifting or breakup [44,83,89], contact metamorphism [14,94], metamorphic decarbonation [82,84], and LIP eruptions [7].

As the carbon budget of convecting mantle is probably shrinking with time [11,59], carbon would be progressively enriched in the lithosphere and the enrichment occurs in two ways. One is the above-mentioned subsurface carbon storage in convergent margins and continental interiors, which occurs prior to the entry of deep carbon into the surface reservoirs. The other is persistent, and more importantly, high-flux carbon sinks to the seafloors through carbonate precipitation and organic carbon burial under the context of elevated deep carbon outflux. High-flux carbon sinks are required to maintain the near steady-state carbon budget of the exogenic system [8]. The well-known deep carbon sources are volcanoes dominated by MORs, plumes, and arcs, which together release ∼53 ± 27 Mt C yr^–1^ to the surface reservoirs (Table 1). Note that the slab-derived fraction of the arc carbon outflux is also considered in the global volcanic CO_2_ emissions because of its deep origins relative to the exogenic system. On the other hand, the deep carbon remobilized during continental reworking in collisional orogens [e.g. 31,95,96] and continental rifts [e.g. 28,29] represents an additional type of deep carbon supply to the surface, which is genetically more related to active faults in non-volcanic regions (i.e. tectonic CO_2_ emissions; [78]).

The most representative collisional orogen on Earth at present is the Tibetan Plateau and its surroundings, i.e. the India-Asia collision zone. A global-scale model of Earth degassing related to extensional tectonics [78] indicates that the India-Asia collision zone is characterized by high probability of tectonic CO_2_ emissions in comparable magnitude with that of the East African rift system. We compiled field-based carbon outflux from the India-Asia collision zone, including the Himalayan fold-and-thrust belt [e.g. 30], northern Himalayan extensional structures [97], as well as southern and southeastern Tibetan Plateau [96]. These estimates yield a total carbon outflux of ∼17–36 (average ∼26) Mt C yr^–1^ for the India-Asia continental collisional orogen (Table 1), which is higher than a recent modeled carbon outflux of ∼5 Mt C yr^–1^ [68] but within the range of 8–84 Mt C yr^–1^ for modern collisional metamorphism [82] (Fig. 6b). Globally, the most well-studied continental rifts with respect to tectonic CO_2_ emissions is the East African rift system [98]. Other extensional tectonics worldwide [78], such as East Asia, North America (i.e. the Basin and Range province), and the circum-Mediterranean regions, have also been investigated [e.g. 66,85,99] but the carbon outfluxes are expected to be lower than the East African rift (Supplementary Data). We suggest a conservative carbon outflux of ∼8–25 (average ∼17) Mt C yr^–1^ (Table 1) for global extensional tectonics, agreeing well with a recent flux estimate of ∼18 ± 14 Mt C yr^–1^ [100] and a conservative estimate of the rift carbon outflux (∼20 Mt C yr^–1^; [83]). Although a higher estimate of 36–124 Mt C yr^–1^ was given in Wong *et al*. [101] for continental rifts, we prefer the conservative estimate in this work because of its coincidence with other estimates (Fig. 6b).

Taken together, it is possible that the collisional orogens and continental rifts could release ∼43 Mt C yr^–1^ (ranging in 26–61 Mt C yr^–1^) to the surface reservoirs. This accounts for ∼45% of the compiled global carbon outflux in this review (∼96 ± 45 Mt C yr^–1^; Table 1) and is surprisingly comparable with volcanic carbon outflux (∼53 ± 27 Mt C yr^–1^) within uncertainties, suggesting the importance of continental reworking in deep carbon recycling. Notably, there is potentially large uncertainty in tectonic CO_2_ emissions because not all of the carbon is of deep origin (e.g. carbon sourced from mantle melting and metamorphic decarbonation at deep crustal levels). The reason for this is that the uprising of CO_2_-rich fluids through active faults is occurring in relatively open systems, which could introduce a considerable amount of shallow carbon (e.g. carbonate dissolution and soil organic carbon) into deeply-sourced carbon [e.g. 51,85,102–104]. Therefore, further subtraction of shallow carbon from the tectonic (or more specifically, diffuse) flux estimate would lower the non-volcanic fraction in global deep carbon outflux. Notably, attention should also be paid to water-gas interaction (e.g. partial exsolution and dissolution of gas in the water) that could result in underestimating the deep CO_2_ output at the surface [51], which suggests that more future work is needed to reduce the uncertainty in tectonic CO_2_ outflux.

### Long-term balance between carbon sources and sinks

Global deep carbon outflux (96 ± 45 Mt C yr^–1^) compiled here is largely comparable to several recent estimates within uncertainty, such as 75–112 Mt C yr^–1^ by Fischer and Aiuppa [98], 76–98 Mt C yr^–1^ by Werner *et al*. [105], and 79 ± 9 Mt C yr^–1^ by Plank and Manning [6]. Obviously, uncertainty would be larger if comparing to an earlier estimate of 174 Mt C yr^–1^ [106]; but this value was questioned to be too high [11]. We note that deep carbon outflux and subduction carbon influx (82 ± 14 Mt C yr^–1^; [6]) are overall balanced within uncertainty. To avoid significant swings in atmospheric CO_2_ levels, the carbon outflux must be balanced by surface carbon sinks over long-term timescales [9,10]. Considering the CO_2_ consumption rate of 47–72 Mt C yr^–1^ by terrestrial silicate weathering [107] and of ∼20 Mt C yr^–1^ by seafloor weathering [18], the geological carbon sources and sinks are roughly balanced within uncertainty over multi-Myr timescales. The organic carbon weathering and burial was assumed to be internally balanced according to the traditional point of view (see [82] and references therein). Notably, a recent study [19] suggests that weathering of rock organic carbon could offset silicate weathering in long-term carbon cycles and requires additional carbon sinks. This is particularly the case when considering deep (i.e. volcanic + metamorphic) and surface (i.e. rock organic carbon weathering) together for the carbon cycling processes in the interface between the lithosphere and Earth's fluid envelope.

### Assessing the global carbon isotope mass balance

**Figure 7.**
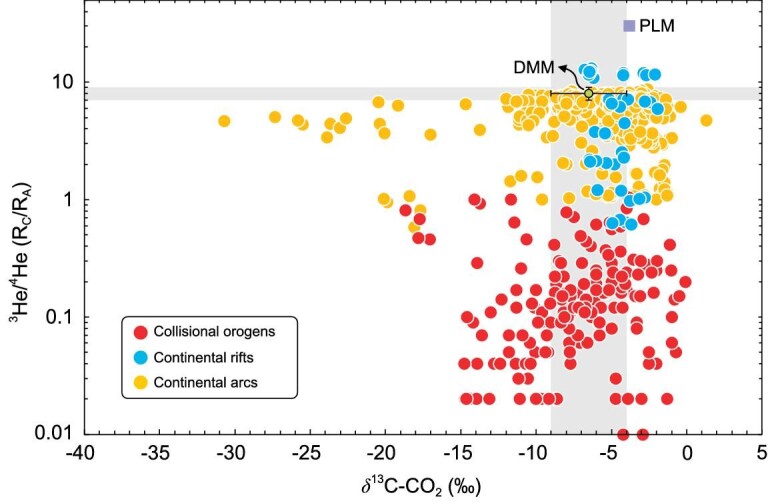
Plot of ^3^He/^4^He vs. δ^13^C-CO_2_ for gas samples from collisional orogens, continental rifts, and continental arcs. Reference values of depleted MORB-source mantle (DMM; [36,109]) and plume mantle (PLM; [126]) are shown for comparison; particularly, the range of ^3^He/^4^He and δ^13^C-CO_2_ is highlighted in gray band for DMM. Data and sources are given in the Supplementary Data.

We compiled ^3^He/^4^He and δ^13^C-CO_2_ data of volcanic and hydrothermal gases from collisional orogens, continental rifts, and continental arcs, together with reference data of DMM and PLM, to constrain the nature of deep carbon entering into the atmosphere and hydrosphere (Fig. 7). Crustal reworking in collisional orogens is characterized by dominant release of crustal carbon (^3^He/^4^He <1 R_A_ and a substantial fraction of the data <0.1 R_A_; Fig. 7) from inorganic and organic sources due to metamorphic decarbonation and water-rock interaction [95]. Spatially, the identified crustal fluids are being predominantly released from the Himalayan orogen and part of southern Tibetan Plateau [108], consistent with metamorphic CO_2_ degassing model related to mountain building [84]. In contrast, continental rifts are dominated by mantle-derived carbon remobilized from the SCLM [44], as evidenced by their high ^3^He/^4^He ratios and mantle-like δ^13^C-CO_2_ values (Fig. 7; [28,29]). Hydrothermal gas samples from continental arcs show large variations in He-C isotopes (Fig. 7), as expected for crustal material recycling into the mantle wedge and potential contamination by overlying crust [109].

Based on estimates of carbon outfluxes from major plate tectonic settings (Fig. 6b) and their corresponding average δ^13^C values constrained by data compilation (Fig. 7), we calculated the average δ^13^C value for solid Earth CO_2_ emissions (i.e. C_input_) into the atmosphere and hydrosphere. This value could be used to evaluate the global carbon isotope mass balance of the present-day Earth, following Mason *et al*. [81], which describes long-term surface CO_2_ sinks (i.e. C_output_) in forms of organic carbon (C_org_) and carbonate (C_carb_) as expressed in Eqns. (1) and (2) [110]:


(1) \begin{eqnarray*}
{\mathrm{\delta }}^{13}{\mathrm{C_{input}\ }} &=& {\mathrm{\ \delta }}^{13}{\mathrm{C_{output}\ }}\\
&=& {\mathrm{\ \delta }}^{13}{\mathrm{C_{org}}}\cdot f_{\mathrm{org\ }} + {\mathrm{\ \delta }}^{13}{\mathrm{C_{carb}}}\cdot f_{\mathrm{carb}}\\
\end{eqnarray*}

(2) \begin{eqnarray*}
f_{\mathrm{org}} + {\mathrm{\ }}f_{\mathrm{carb}} = {\mathrm{\ }}1
\end{eqnarray*}

where input and output denote CO_2_ release into and removal from the atmosphere and hydrosphere; *f*_org_ and *f*_carb_ are the proportion of surface carbon that is sequestered through organic carbon (δ^13^C_org_ = –30‰) burial and carbonate (δ^13^C_carb_ = 0‰) precipitation, respectively.

The calculated results show that the flux-weighted average δ^13^C value of modern global deep carbon emissions (i.e. δ^13^C_input_) may be about –5.5‰, which corresponds with a slightly lower fraction of organic carbon burial (*f*_org_ = 0.18) than the canonical *f*_org_ value of 0.2 [110]. In other words, our results suggest that the fraction of surface CO_2_ sinks as organic carbon may be smaller than previously assumed, consistent with the estimate by Mason *et al*. [81]; however, more work is needed to reduce the uncertainties. Generally, the potential biased results could be caused by erroneous estimates of deep carbon outfluxes and unrepresentative selection of average δ^13^C values for different plate tectonic settings (i.e. MORs, plumes, arcs, orogens, and rifts; Fig. 6b). For example, the shallow carbon fraction was not subtracted from total carbon outfluxes of collisional orogens and continental rifts in our calculation (Fig. 6b), due to the lack of quantitative evidence for a global scale assessment of shallow carbon emissions in these tectonic settings.

### Uncertainty evaluation

The uncertainties in carbon mass balance assessment could exist in each step of calculation concerning the carbon influx at subduction zones and carbon outflux from global plate tectonic settings. Our illustration about the uncertainties was given in the above discussion where necessary. We note that previous reviews [e.g. 5,6,11] also pointed out the influence of various subducting carbon assemblages from trench to trench on the quantification of carbon recycling efficiency. In addition, the methods of carbon outflux estimation have different uncertainties. Also inevitable is that extrapolation of regional carbon flux estimates to global scales is highly uncertain. We further emphasize that current studies are insufficient to reconcile the spatial heterogeneity of deep carbon recycling in the context of global plate tectonics. For example, the proportion of carbon released outside of volcanic arcs (i.e. fore-arcs and back-arcs) is not as well-constrained as that has been done for the arc volcanoes [55]. Globally, many tectonically active regions are still uninvestigated for deep carbon outflux [78]. Therefore, challenges remain for future research.

## CHALLENGES AND FUTURE RESEARCH OPPORTUNITIES

As one of the most frontier research topics in Earth and environmental sciences, carbon cycle and Earth's habitability have been extensively studied over the past few decades, with the significant advances boosting our understanding of (i) the reservoirs, fluxes, and mechanisms of deep carbon recycling from a global mass balance point of view, and (ii) the interaction between deep and surface carbon reservoirs and its impact on Earth's surface environment and the physico-chemical properties of its interior. Despite recent advances, several important aspects remain ‘the limits to knowledge’ and may guide future research.

### Toward a full chain of carbon recycling path: quantifying the mobilization, transport, and fluxes of carbon from sources to sinks

A complete picture of carbon recycling in convergent plate margins refers to carbon transfer between the deep and surface reservoirs following a recycling path of source-to-sink-to-source. It mainly includes subduction-driven carbon inputs to the mantle, carbon mobilization at fore-arc to sub-arc depths, trans-lithospheric magma transfer to the surface, CO_2_ outgassing, and subsequent CO_2_ removal from the atmosphere and oceans through carbonate precipitation and organic carbon burial [e.g. 5,6,8]. Each step of the carbon recycling path must be constrained following a quantitative workflow that integrates field-based observations, experiments, and thermodynamic modeling. Zooming into the subduction channel, the carbon behavior along subduction geotherms determines the capacity of subducting slab in transferring carbon to mantle wedge and convecting mantle beyond sub-arc depths [11]. Although attempts have been made to constrain carbon loss from the subducting slab and its storage at fore-arc and sub-arc depths [e.g. 11,55,57,59], the heterogeneity in global subduction zones remains less understood. Another important question is the replenishment efficiency of the convecting mantle by subducted carbon [57]. For CO_2_ output from arc volcanoes, future quantification work could focus on both carbon loss from the ascending magmas [11] and carbon addition due to contact metamorphism at crustal depths [e.g. 14,81,94]. Viewed from global plate tectonics, how the spatial heterogeneity in deep CO_2_ emissions could be incorporated into carbon cycle models should be further refined [6]. Overall, many questions are still open and require future research to establish a full chain of carbon recycling path.

### Integrating plate margins with the intra-plate: understanding deep carbon recycling from a whole-Earth dynamics point of view

Deep carbon recycling is operating in the regime of global plate tectonics. The plate margins are expected to interact dynamically with intra-plate settings, leading to regional- to global-scale expression in geophysical, geochemical, and geological evolution. A classic example for how plate marginal processes could influence intra-plate evolution (and more importantly the deep CO_2_ emissions) is destruction of the North China Craton (NCC). It is widely accepted that subduction of the paleo-Pacific plate played a dominant role in destruction or decratonization of the NCC in the Mesozoic [e.g. 63,111], which highlights the impact of oceanic plate subduction on the cores of continents at a global scale [111]. By triggering pervasive metasomatism of the SCLM [112] and addition of deeply subducted slab materials into the big mantle wedge [64,113], the successive subduction of the paleo-Pacific plate and Pacific plate beneath East Asia since early Jurassic [63] has the potential to release huge amounts of deep CO_2_ into the atmosphere. This mechanism has been recently highlighted for the destruction stage of the NCC [114] and the big mantle wedge characterized by deep subduction-driven recycling of carbon-rich slab components [64]. Future research remains open for quantification of deep CO_2_ outfluxes in the geological past (such as the magmatism during and postdating the cratonic destruction period; [114,115]), as well as that still ongoing via Cenozoic volcanoes above the present-day big mantle wedge [65,66].

The importance of continental plate interiors, such as the NCC and other ancient cratons, lies also in their capacity to retain huge amounts of carbon-rich components over its prolonged history [44] and their potential as a globally significant carbon source [28,29,83]. Therefore, reworking of the SCLM (especially the cratonic mantle) during continental rifting, together with the crustal reworking that occurs in different tectonic regimes but features particularly in continental collision zones [17,80], should be considered as important as deep carbon processes at MORs, arcs, and plumes. In future modeling of deep carbon cycle and climate change targeted on supercontinent cycles [10], the dynamic interaction between plate margins and interiors must be well constrained in terms of CO_2_ sources and sinks. The plate-tectonics–based deep carbon dynamics [24], together with potential mantle plume perturbations [7], should be integrated in global carbon cycle models, which may guide future research on deep-time reconstruction of deep carbon cycles.

### Standing between the past and future: deep-time reconstruction and future-oriented modeling of global carbon cycle

The rapidly increasing atmospheric CO_2_ levels (∼422 parts per million by volume; as of December, 2023) have exerted considerable threats to the sustainability of the Earth's habitable conditions. To understand what is happening today, it is informative to give a retrospect on the past. Likewise, our understanding of the past and present would shed light on Earth's future. Numerical models have thus been established for reconstruction of global carbon cycles through deep time, as best exemplified by the landmark Berner-Lasaga-Garrels (BLAG) model [116]. Owing to the advances achieved over years, the refined models of plate tectonic reconstruction [117] and paleogeography configuration [118], and our growing knowledge of carbon reservoirs and fluxes [e.g. 6,8,11] as well as carbon recycling mechanisms [e.g. 6,14,58], have allowed a better understanding of the controls on atmospheric CO_2_ levels and global climate changes. A recent review by Müller *et al*. [24] presents an example for reconstructing the plate-tectonics–based deep carbon cycle through geological time in a source-driven framework. In addition, Zhao *et al*. [119] proposed a conceptual workflow for numerical modeling that integrates plate tectonics and deep carbon dynamics, which would guide future research on tectonic carbon cycle modeling.

Notably, uncertainties remain in terms of (i) the reliability of reconstructed deep-time Earth, and (ii) the complex boundary parameters that are, in many cases, less constrained for numerical models. Taking into account geological records (e.g. terrestrial and marine proxies) that are used to reconstruct the atmospheric CO_2_ concentration and global average temperature in the past [120], numerical modeling could track deep carbon cycle and its climatic impacts with relatively high confidence over the Phanerozoic [121] (e.g. the Mesozoic and Cenozoic in particular [24,83,101]) or into the Neoproterozoic [122]. Clearly, the uncertainty would increase for numerical modeling deeper into Earth's history due to the sparse preservation of geological records. Additionally, some critical boundary parameters in numerical models remain loosely constrained, such as the temporal and spatial heterogeneity in deep CO_2_ outfluxes from continental rifts [83] and collisional orogens [95]. In particular, the spatially heterogeneous carbon recycling efficiency, as a function of slab carbon-bearing components and subduction-zone thermal regimes [5,6], must be further refined to match the reconstructed deep-time convergent plate margins and related deep carbon recycling processes. Overall, extrapolation to deeper time intervals and/or to wider spatial scales, especially integrating carbon cycle dynamics in different plate tectonic settings (and thus various modeling parameters), would introduce uncertainty to the reconstruction results, which remains challenging but intriguing to boost future research.

The modeling of near-future global changes has been well studied for different scenarios of CO_2_ emissions in the Anthropocene [123]. Toward the scenarios of the evolving system of global plate tectonics (e.g. the next supercontinent—Pangea Ultima), an emerging research topic related to critical life-essential volatile elements pays attention to the fate of Earth's habitability, as shown in recent work by Farnsworth *et al*. [124]. Similar to reconstruction of the past global carbon cycle [24], a general consideration in modeling the future deep carbon cycles should include deep CO_2_ outgassing rates of different convergent plate margins, mid-ocean ridges, intra-plate settings (e.g. continental rifts), plateau uplift and orogenic processes (e.g. crustal reworking and continental weathering), the position of arc-continent collision zones, and so on [119]. Combining these controlling factors and beyond, the interaction between CO_2_ sources and sinks could be evaluated to gain a complete picture of global deep carbon cycles, and particularly, to constrain how Earth's life-fostering layers would evolve during future climate changes to maintain or lose the habitable conditions for various life forms.

## Supplementary Material

nwae089_Supplemental_File
